# Comment on ‘A Prospective Observational Study to Assess the Surgical Site Infections Following Abdominal Surgeries in a Tertiary Care Teaching Hospital’

**DOI:** 10.1002/hsr2.71413

**Published:** 2025-10-22

**Authors:** Raza Ur Rehman, Ahmad Furqan Anjum

**Affiliations:** ^1^ Shaikh Khalifa Bin Zayed Al Nahyan Medical and Dental College Lahore Punjab Pakistan

## Transparency Statement

The lead author (Raza Ur Rehman) affirms that this letter is an honest, accurate, and transparent account of the commentary being provided; no important aspects have been omitted. All authors have read and approved the final version of this letter. The corresponding author (Raza Ur Rehman) takes full responsibility for the accuracy and integrity of its content.

Dear Editor,

I enthusiastically read the article “A Prospective Observational Study to Assess the Surgical Site Infections Following Abdominal Surgeries in a Tertiary Care Teaching Hospital” by Sharma et al., published in Health Science Reports (21 July 2025; Volume 8, Issue 7; https://doi.org/10.1002/hsr2.71095). This timely investigation provides valuable insights into the burden of surgical site infections (SSIs) in a resource‐constrained Indian healthcare setting. It highlights both incidence trends and institutional risk factors. The study′s prospective design and focus on abdominal surgeries make it a significant contribution to regional understanding of SSIs. Importantly, the authors link infection surveillance data with preventive strategies, offering practical directions for infection control and benchmarking. Their findings underscore the urgent need to contextualize infection metrics in low‐ and middle‐income countries (LMICs), where infrastructural limitations and epidemiological patterns differ substantially from those in high‐income nations.

I would like to draw your attention to an important perspective that the study has missed. Firstly, the manuscript interprets prolonged hospital stay as a causal factor for SSI development. However, robust evidence suggests that SSIs themselves often prolong hospital stays, not the other way around. Without temporal modeling, such associations may reflect reverse causality, leading to faulty conclusions and misdirected interventions. Traditional logistic models fail to account for time‐varying confounders, particularly in hospital‐acquired conditions. This results in biased associations where infection appears to be predicted by variables it may actually cause [1]. Infections like SSIs do not merely co‐occur with long stays—they often prolong them. Adjusted models reveal that much of the increased stay previously attributed to pre‐existing factors is actually driven by the infection event itself [2]. Secondly, The study reports overall SSI incidence but does not stratify or analyze outcomes by infection depth (superficial, deep, or organ‐space). This omission is critical, as these subtypes differ in etiology, severity, risk factors, and outcomes. Superficial infections are often self‐limited, while organ‐space infections carry a high risk of sepsis and reoperation. Without clear stratification, interventions may be poorly matched to risk [3]. Moreover, systemic patient‐related factors—specifically diabetes (both insulin‐ and non‐insulin‐dependent), malnutrition (evidenced by significant preoperative weight loss), and postoperative anemia—are well‐described independent predictors of surgical site infection [4]. There is considerable heterogeneity in SSI predictors depending on the depth of infection. Targeted interventions require granular surveillance data, not generalized SSI categories [5].

Thirdly, the article overlooks the influence of seasonal and climatic variations (temperature, humidity, and monsoon cycles) on SSI incidence. This confounding factor critically undermines inter‐facility comparison, particularly in diverse climates. Across 14 studies, it is observed that warmer months were associated with a 29% increased risk of SSI. The absence of seasonal stratification in surveillance programs leads to flawed institutional benchmarking [6]. July through September recorded the highest infection rates, even after adjustment for case mix. Temperature and seasonality are non‐randomized confounders in SSI surveillance [7]. Meteorological trends strongly predict SSI distribution. Ignoring climatic differences compromises cross‐region epidemiologic validity [8]. Fourthly, the article implies institutional safety protocols primarily explain differences in SSI rates. However, hospital case mix, referral bias, patient acuity, and resource limitations are crucial unmeasured variables. Institutional comparisons must adjust for case complexity and referral bias. Protocol adherence explains only a portion of the infection risk variance [9]. Without granular adjustment for staff‐to‐patient ratios, equipment quality, and infrastructure, attributing differences to safety adherence is premature [10]. While the interference factors proposed by Sharma et al. are important, future clinical studies should move beyond description and actively stratify these variables during experimental design. I recommend grouping factors into domains such as patient‐level comorbidities (e.g., diabetes, obesity, immunosuppression), procedural variables (e.g., clean vs. contaminated surgery, operative duration), and perioperative management (e.g., timing of antibiotic prophylaxis, use of wound care bundles). Employing multilevel and multivariable modeling, as demonstrated in a large cohort study identifying patient‐, ward‐, and hospital‐level contributors to SSI risk, can help disentangle overlapping effects and enhance predictive clarity [11]. Structuring data collection and analysis in this way will not only facilitate more reliable risk prediction but also support targeted preventive interventions and refine the design of subsequent trials. More recent data also suggest climate change will exacerbate these seasonal trends, with rising global temperatures projected to increase SSI incidence and complicate prevention strategies [8].

Lastly, the article omits the role of AMR profiles in infection severity, treatment complexity, and failure rates. Resistance patterns vary by region and significantly influence outcome disparities and length of stay. SSIs caused by resistant organisms result in longer hospitalization and higher mortality. Surveillance without AMR profiling lacks clinical resolution [12]. Kaye, K.S. et al. (2014) study shows the prevalence of MRSA and ESBL‐producing bacteria in SSIs undermines generalizations. AMR data must be a part of infection reporting [13]. Hospitals facing high endemic resistance cannot be evaluated by the same SSI baselines as others. Adjustment is not just ideal but essential [14]. Recent surveillance highlights this concern: WHO's 2022 AMR report emphasizes rising resistance in common SSI pathogens, with global prevalence of carbapenem‐resistant *Klebsiella pneumoniae* and MRSA undermining standard treatment options [15].

In conclusion, while Sharma et al.'s study offers valuable insights into SSI trends in an Indian tertiary care context, the interpretation of risk factors would benefit from deeper methodological and contextual refinement. Specifically, distinguishing causation from correlation, stratifying infection types, accounting for seasonal variation, and incorporating antimicrobial resistance data are essential steps toward more precise and actionable findings. Future research should adopt more granular analytical frameworks and adjust for structural disparities and environmental factors. These refinements will not only enhance the scientific validity of such studies but also strengthen their relevance for public health policy and clinical practice in low‐resource settings.

## Author Contributions

**Raza Ur Rehman:** conceptualization, investigation, visualization, writing – original draft, writing – review and editing, supervision, data curation. **Ahmad Furqan Anjum:** writing – review and editing, formal analysis, validation.

## Ethics Statement

This article is a Letter to the Editor and does not involve new patient recruitment, statistical analysis, or experimental procedures. Therefore, ethical approval, patient consent, and CONSORT reporting are not applicable.

## Conflicts of Interest

The authors declare no conflicts of interest.

## Data Availability

Data sharing not applicable to this article as no datasets were generated or analyzed during the current study.
